# Critical role of SIK3 in mediating high salt and IL-17 synergy leading to breast cancer cell proliferation

**DOI:** 10.1371/journal.pone.0180097

**Published:** 2017-06-28

**Authors:** Suneetha Amara, Ciera Majors, Bipradas Roy, Salisha Hill, Kristie L. Rose, Elbert L. Myles, Venkataswarup Tiriveedhi

**Affiliations:** 1 Department of Medicine, St Thomas-Midtown, Nashville, Tennessee, United States of America; 2 Department of Biological Sciences, Tennessee State University, Nashville, Tennessee, United States of America; 3 Mass Spectrometry Research Center, Vanderbilt University, Nashville, Tennessee, United States of America; 4 Department of Biochemistry, Vanderbilt University, Nashville, Tennessee, United States of America; 5 Department of Cancer Biology, Vanderbilt University, Nashville, Tennessee, United States of America; University of South Alabama Mitchell Cancer Institute, UNITED STATES

## Abstract

Chronic inflammation is a well-known precursor for cancer development and proliferation. We have recently demonstrated that high salt (NaCl) synergizes with sub-effective interleukin (IL)-17 to induce breast cancer cell proliferation. However, the exact molecular mechanisms mediating this effect are unclear. In our current study, we adopted a phosphoproteomic-based approach to identify salt modulated kinase-proteome specific molecular targets. The phosphoprotemics based binary comparison between heavy labelled MCF-7 cells treated with high salt (Δ0.05 M NaCl) and light labelled MCF-7 cells cultured under basal conditions demonstrated an enhanced phosphorylation of Serine-493 of SIK3 protein. The mRNA transcript and protein expression analysis of SIK3 in MCF-7 cells demonstrated a synergistic enhancement following co-treatment with high salt and sub-effective IL-17 (0.1 ng/mL), as compared to either treatments alone. A similar increase in SIK3 expression was observed in other breast cancer cell lines, MDA-MB-231, BT20, and AU565, while non-malignant breast epithelial cell line, MCF10A, did not induce SIK3 expression under similar conditions. Biochemical studies revealed mTORC2 acted as upstream mediator of SIK3 phosphorylation. Importantly, cell cycle analysis by flow cytometry demonstrated SIK3 induced G0/G1-phase release mediated cell proliferation, while SIK3 silencing abolished this effect. Also, SIK3 induced pro-inflammatory arginine metabolism, as evidenced by upregulation of the enzymes iNOS and ASS-1, along with downregulation of anti-inflammatory enzymes, arginase-1 and ornithine decarboxylase. Furthermore, gelatin zymography analysis has demonstrated that SIK3 induced expression of tumor metastatic CXCR4 through MMP-9 activation. Taken together, our data suggests a critical role of SIK3 in mediating three important hallmarks of cancer namely, cell proliferation, inflammation and metastasis. These studies provide a mechanistic basis for the future utilization of SIK3 as a key drug discovery target to improve breast cancer therapy.

## Introduction

Chronic inflammation is a well-known precursor for cancer development and proliferation [1]. Unlike acute inflammation which exerts a beneficial pathogen or disease eliminatory function, chronic inflammation initiates a cascade of molecular events that causes malignant transformation of terminally differentiated cells and thus leading to cancer development. These smoldering chronic inflammatory events induce reactive oxygen and nitrogen species (RNS/ROS) and thus resulting in DNA damage and tumor formation. Along with this, chronic inflammation is known to induce a series of signaling transcription factors which promote uncontrolled cell division and tumor progression. The cellular stress caused by inflammation induces release of several growth factors which induce neo-vascularization to the tumor. Cancer cells metastasize through these newly formed blood vessels to various parts of the body [2].

Tumor microenviroment has several inflammatory cytokines and chemokines that have been shown to mediate the progression and proliferation of cancer [3]. One of the cytokines that has evoked a lot of recent research interest is Th17 lineage specific cytokine, interleukin (IL)-17, which has been shown to have a dual, tumor-elimination and tumor progression effect [4]. It is of interest to note that high salt (sodium chloride, NaCl) induces a Th17 differentiation of naïve CD4+T-cells [5]. While the exact role of salt in cancer is unclear, recent studies from our laboratory have demonstrated that high salt (50 mM above basal conditions) synergized with sub-effective concentration of IL-17 (0.1 ng/mL) to induce cancer cell proliferation, RNS/ROS release, and pro-angiogenic VEGF secretion [6, 7]. Importantly, sodium-MRI studies in breast cancer patients have demonstrated an increased sodium content, of up to 63% above the surrounding soft tissue, in the breast tumors [8, 9]. All these studies support a possible notion that high salt exerts an effector role on tumor progression, either working individually or synergistically to enhance an inflammatory tumor microenvironment.

Traditionally, high osmolality in the tumor and lymph node microenvironment is suggested to induce cellular activation [10]. However, various osmotic stress induced inflammation studies in cancer have demonstrated that exceptionally high concentration (0.5 to 6 moles/Liter) of solutes (such as mannitol, sorbitol, urea) is needed to induce cellular activation [10, 11]. Previous studies in our laboratory have demonstrated that a modest 50 mM increase in NaCl concentration was sufficient to induce pro-cancer cellular responses [6]. Importantly, unlike NaCl, equimolar mannitol (50 mM mannitol) was not able to induce similar cellular responses. Interestingly, other labs have also shown that similar concentration of NaCl was sufficient to induce T-Lymphocyte activation [5]. To date, very little is known on the signaling mechanisms mediated by high salt to induce pro-cancer effect. In our current study, using phospho-proteomics approach, we have identified a novel salt specific kinase, salt-inducible Kinase-3 (SIK3), to play a critical role in mediating high salt induced inflammatory signaling responses leading to cancer cell proliferation.

## Materials and methods

### Cell cultures and plasmids

Five breast tissue related cell lines were used in our studies, of these, four breast cancer cells (MCF7, MDA-MB-231, BT20, AU565) and one non-malignant breast epithelial cell line (MCF10A) were utilized and obtained from the American Type Culture Collection (ATCC, Manassas, VA) The cells were cultured in cell basal essential media (RPMI1640 media, Thermo Fisher Scientific, Waltham, MA) along with the media supplements such as fetal bovine serum, penicillin/streptomycin, fungizone, HEPES and glutamine, as recommended by the manufacturer and as previously described [6, 12]. Cell lines were frozen in liquid vapor nitrogen at -130°C until use. Upon thawing, cells were maintained in 5% CO_2_ incubator in sterile essential media at 37°C. For salt and interleukin-17 treatment conditions, cell culture media was supplemented with 0.05 M NaCl (Sigma Aldrich, St Louis, MO) and 0.1 ng/mL IL-17 (Life Technologies, Grand Island, NY). We have previously performed a dose-response for salt (0–0.1 M NaCl) and IL-17 (0–1000 ng/mL) and found-out that 0.05 M NaCl provided highest cell proliferation [6, 13] and 0.1 ng/mL of IL-17 induced sub-effective inflammatory cytokine response [6, 14]. All chemicals unless mentioned were obtained either from Sigma Aldrich (St Louis, MO) or Thermo Fisher Scientific (Waltham, MA). Tagged SIK3 cDNA plasmid was obtained from Origene (Rockville, MD) in pCMV6 vector. The obtained SIK3 cDNA is cloned to pcDNA3.1 with SfaAI and MluI restriction sites at 5’ and 3’ ends, respectively. The following primers were used for cloning SIK3: forward: 5’-GCGATCGCCATGCCCGCCCGTATCGGCTAC-3’ and Reverse: 5’-ACGCGTCACGCCTGCCTGCTCCATGCTGAAGG-3’. For mutant, SΔA493-SIK3 cloning the following site direct mutagenesis primers were used: mutant downstream primer: 5’-CGGAGGGCAGCAGATGGAGGAGCCAAC-3’ and wild-type upstream primer (reverse complement): 5’- GCCAAGGGGGCCCATTCCATTCAACAG-3’. The site directed mutagenesis is performed using Q5^R^-site directed mutagenesis kit (New England BioLabs, Ipswich, MA). All other cloning reagents, restriction enzymes and related buffers were obtained from New England BioLabs (Ipswich, MA). For siRNA knock down of SIK3 we have used the following two siRNA sequences: SIK3-siRNA-1: 5’-GUGCAGAGUGUUGGAGUCC -3’; SIK3-siRNA-2: 5’-UGAUAAGAUAAAGCCUGGC-3’; scramble SIK3-siRNA-1: 5’- UGGAGGCGAGUCAGUUUGC-3’; scramble SIK3-siRNA-2: 5’- GUAAAGCGAUUAACCAGUG-3’. The siRNA for RICTOR and RAPTOR along with scramble controls were commercially obtained (Santa Cruz Biotech and Thermo Fisher Scientific).

### Phospho-proteomic analysis

For SILAC-labelling of MCF-7 cells the cells were cultured for 7 cycles in heavy and light labelled lysine and arginine. All reagents for phospho-protemics study including conditioned media, supplements, heavy and light arginine/lysine were obtained from Thermo Fisher Scientific (Waltham, MA). The incorporation of heavy residues was confirmed by mass spectrometry at Vanderbilt proteomics core facility (Nashville, TN). Following confirmation of label incorporation, cells were treated under basal conditions for light labeled cells and with 0.05 M NaCl supplemented media for heavy labeled cells. Both cells were to 80–90%confluence in multiples of T175 flasks to obtain a 2 gm of total protein individually or light and heavy labeled cells. The cells were washed with cold phosphate buffer saline (PBS) and were detached using trypsin/EDTA. The cells were then pelleted at by centrifugation and supernatants were removed. The cell pellets were latter lysed with RIPA buffer (150 mM NaCl, 50 mM Tris and 1% NP-40, 0.25% sodium deoxycholate, 1 mM EDTA, pH8.0), with Halt protease inhibitor cocktail (Thermo Fisher Scientific, Waltham, MA), and Phosphatase inhibitor cocktail 3 (Sigma Aldrich, St Louis, MO) for 30 minutes on ice and lysates were clarified by centrifugation at 17,000 x *g*, at 4°C. The total protein concentration was quantified using a Bradford Protein Assay Kit (Thermo Fisher Scientific, Waltham, MA) as per the manufacturer’s protocol. Aliquots were taken for western blotting. Prior to protein digestion, trifluoroethanol was added to the combined SILAC protein lysates for a final concentration of 50% trifluoroethanol. Proteins were reduced by addition of 0.5 mol/L Tris [2-carboxyethyl] phosphine for 1 hour at room temperature, and carbamidomethylation was completed by treatment with iodoacetamide for 30 minutes in the dark at room temperature. Proteins were digested overnight at 37°C with proteomics-grade trypsin. The resulting peptides were desalted by solid-phase extraction. Digested samples first were acidified and diluted with 0.1% trifluoroacetic acid, and loaded onto the Sep-pak solid-phase (Waters, Milford, MA) extraction material. After sample loading, the cartridges were washed with 0.1% trifluoroacetic acid, and peptides were eluted with 60% acetonitrile in 0.1% trifluoroacetic acid. Three sequential elutions were performed, and eluates were reduced to dryness via vacuum centrifugation.

Phosphopeptides were enriched as previously described, but with minor modifications [15]. Purified tryptic peptides were resuspended in buffer A (0.05% heptafluorobutyric acid/2% acetonitrile) containing 300 mg/mL lactic acid (buffer B). Titanosphere titanium oxide 5-μm beads (GL Sciences, Tokyo, Japan) were used for each SILAC analysis. The beads were first washed with buffer C (0.05% heptafluorobutyric acid/80% acetonitrile), and then added to the resuspended tryptic peptides. The mixtures were rotated at room temperature for 30 minutes, centrifuged, and the supernatant was discarded. The phosphopeptide-bound beads were washed with buffer D (buffer C containing lactic acid), and then twice with of buffer C. Phosphopeptides were eluted from the titanium oxide beads using 0.5 mol/L ammonium hydroxide and then twice with 5 mol/L ammonium hydroxide before drying by vacuum centrifugation. Each elute was reconstituted in 0.1% formic acid.

Phosphopeptides were loaded onto a self-packed biphasic C18/SCX MudPIT column using a Helium-pressurized cell (pressure bomb). The MudPIT column consisted of 360 x 150um i.d. fused silica, which was fitted with a filter-end fitting (IDEX Health & Science) and packed with 6cm of Luna SCX material (5um, 100Å) followed by 4cm of Jupiter C18 material (5 um, 300Å, Phenomenex). Once the sample was loaded, the MudPIT column was connected using an M-520 microfilter union (IDEX Health & Science) to an analytical column (360um x 100um i.d.), equipped with a laser-pulled emitter tip and packed with 20cm of C18 reverse phase material (Jupiter, 3um beads, 300Å, Phenomenex). Using an Eksigent NanoLC or Thermo Scientific Easy nanoLC and Autosampler, MudPIT analysis was performed with an 8-step salt pulse gradient (25, 50, 75, 100, 150, 250, 300, 500, 750, and 1 M ammonium acetate). Following each salt pulse, peptides were gradient-eluted from the reverse analytical column at a flow rate of 500nL/minute, and the mobile phase solvents consisted of 0.1% formic acid, 99.9% water (solvent A) and 0.1% formic acid, 99.9% acetonitrile (solvent B). For the peptides from the first 7 SCX fractions, a 120-min reverse phase gradient was used consisting of 2–40% solvent B in 105min followed by a 15 minute equilibration at 2% solvent B. For the last (1M salt) fraction, the peptides were eluted from the reverse phase analytical column using a gradient of 2–98% solvent B in 105 minutes. Peptides were introduced via nano-electrospray into a LTQ Orbitrap Velos mass spectrometer (Thermo Scientific, Waltham, MA), and the data were collected using a 17-scan event data–dependent method. Full scan (m/z 350–2000) spectra were acquired with the Orbitrap as the mass analyzer (resolution, 60,000), and the 16 most abundant ions in each MS scan were selected for fragmentation in the ion trap. An isolation width of 2 m/*z*, activation time of 10 ms, and 35% normalized collision energy were used to generate tandem mass spectrometry spectra.

For peptide and protein identification, data were analyzed using the Maxquant software package, version 1.3.0.5 [16]. MS/MS spectra were searched against a human subset database created from the UniprotKB protein database (http://www.uniprot.org). Precursor mass tolerance was set to 20 ppm for the first search, and for the main search, a 10-ppm precursor mass tolerance was used. The maximum precursor charge state was set to 7. Variable modifications included carbamidomethylation of cysteines (+57.0214), oxidation of methionines (+15.9949) and phosphorylation of serine, threonine and tyrosine (+79.9663). Enzyme specificity was set to Trypsin/P, and a maximum of two missed cleavages were allowed. The target-decoy false discovery rate (FDR) for peptide and protein identification was set to 1% for peptides and 2% for proteins. A multiplicity of 2 was used, and Arg10 and Lys8 heavy labels were selected.

### Western blot/ Immunoprecipitation

Total proteins were extracted from cells with lysis buffer for Western blot analysis as previously described [17, 18]. Total proteins were separated on a 4–12% sodium dodecyl sulfate-polyacrylamide gradient gel and transferred onto a nitrocellulose membrane. The membranes were blocked overnight at 4°C in Tris-buffered saline with 0.05% Tween 20 (5% nonfat milk in 10mM Tris-HCl-100mM NaCl-0. 1% Tween 20, pH 7.4). The membranes were incubated first with Abs specific for total and phosphorylated forms at room temperature with primary Abs diluted 1 in 1,000 in blocking buffer for 2hrs, and then with a horseradish peroxide-conjugated secondary IgG mAb diluted 1 in 5,000 for 1hr. All primary and secondary Abs were obtained from Santa Cruz Biotech (Dallas, TX). All primary and secondary antibodies for Western blot and immunoprecipitation were obtained from either AbCam (Cambridge, MA) or Santa Cruz Biotech (Dallas, TX). The following specific primary antibodies to SIK3 (ab211424, AbCam) GADPH (sc-47724, Santa Cruz), Actin (sc-8432, Santa Cruz), HDAC4 (sc-46672, Santa Cruz), pHDAC4-S632 (ab39408, AbCam), AKT (sc-135829, Santa Cruz), pAKT-S473 (sc-293125, Santa Cruz), S6K1 (ab9366, AbCam), pS6K1-T389 (ab2571, AbCam), RICTOR (sc-271081, Santa Cruz), RAPTOR (sc-81537, Santa Cruz), iNOS (sc-7271, Santa Cruz), Arg-1 (sc-4496 WB, Santa Cruz), ASS-1 (sc-365475, Santa Cruz) and ODC (sc-398116, Santa Cruz). The membrane was developed using the chemiluminescence kit (Millipore) and analyzed on using Bio-Rad Universal Hood II (Hercules, CA). Morphometric analysis was done using the software provided by the company.

For SIK3 immunoprecipitation, the cultured cells were washed with cold PBS, and lysed for 30 min on ice with 0.5 mL of lysis buffer as previously mentioned [14, 19]. To the lysis buffer 0.5 mL of dilution buffer was added and centrifuged at 17,000 x g for 30 min. The supernatant was transferred and 1 μg normal chicken IgY (ab97135, AbCam) or chicken anti-SIK3 were added. After overnight incubation at 4°C, 30 μL carbolink beads (Pierce, Rockford, IL) were added to lysates and incubated for 2 hours for chicken antibody immunoprecipitation as per manufacturer’s protocol. Beads were washed with 700 μL of wash ice cold buffer four times, 3 min each time followed by centrifugation at 1,800 x g for 3 min at 4°C. Beads were then washed with cold PBS and bound proteins were eluted by boiling with 30 μL of 2X SDS buffer for 10 min. Proteins were subjected to SDS-PAGE (4–12% gel) and immunoblotting. Phosphorylation of SIK3 were detected with a mouse monoclonal phospho-serine antibody (sc-81514, Santa Cruz).

### Quantitative reverse transcription polymerase chain reaction

Expression profiles of genes at mRNA level in the breast cancer cell lines were analyzed using the TaqMan FAM-labeled RT-PCR primers for SIK3 (Hs00228549_m1), GADPH (Hs402869), and Actin (Hs4333762T), obtained from Applied Biosystems/Thermo Fisher Scientific (Grand Island, NY) as per the manufacturer’s recommendation. Briefly, total RNA was extracted from 10^6^ cells using TRIzol reagent (Sigma–Aldrich, St Louis, MO) and analyzed as mentioned previously [7, 20, 21]. RNA samples were quantified by absorbance at 260nm. The RNA was reverse-transcribed and RT-PCR (real time PCR) was performed in a final reaction volume of 20 μL using BioRad CFX96 (Hercules, CA). Each sample was analyzed in triplicate. Cycling conditions consisted of an initial denaturation of 95°C for 15min, followed by 40 cycles of 95°C for 30s, followed by 61°C for 1min.

### Cell proliferation assay

Cell viability was measured by trypan blue dye exclusion (Sigma Aldrich, MO) and MTT assay (Life technologies, CA) as previously described [6]. Briefly, the viability of breast cancer cells was assessed by measuring mitochondrial activity using MTT (4,5-dimethylthiazol-2-yl)2,5-diphenyltetrazolium bromide) assay. For various treatment conditions the cancer cells were plated in 96 well plate for 48–72 hours, the cells were incubated with 5mg/mL MTT in PBS for 2 hrs, latter lysed with manufacturer provided reagents and read at 562 nm. Viability was calculated as percentage compared to untreated cells.

### HDAC4 assay kit

The HDAC4 activity analysis (Epigentek, Farmingdale, NY) was performed on the nuclear fragments of the cell lysates under various assay conditions as per manufacturer’s instructions. Colorimetry detection at 450 nm was performed using EMax Plus spectrophotometer and data analysis was carried out using software provided by the manufacturer (Molecular Devices, Sunnyvale, CA). The data analysis was performed based on a standard curve obtained using the positive controls provided by the manufacturer as previously described.

### Cell cycle assay

For cell cycle assay, cells were washed with ice cold PBS and were latter resuspended in 70% ethanol and then incubated at −20°C overnight. The cells were then incubated with PBS containing Propidium Iodide (PI, 40 μg/ml) and 0.25 μg/mL RNase A for 30 min. The PI-stained cells were determined with a FACS Calibur/LSRII flow cytometer (Becton-Dickinson, Franklin Lakes, NJ). Results were analyzed with the BD FACSDiva software software. For G0/G1 synchronization of the cell cycle, the cells were grown to 40–50% confluency and then serum starved for 48 hours later the cells were washed and cultured with regular media under various treatment conditions for another 48 hours. For G2/M-phase synchronization nocodazole 100 ng/mL (Sigma Aldrich, St Louis, MO) for 24 hours at 40–50% confluency, latter washed and cultured under various treatment conditions for analysis. For S-phase synchronization cells were first cultured to 40–50% confluency with complete media, latter washed and serum starved for 24 hours following which cultured in complete media with aphidocolin (50 ng/mL, Sigma Aldrich, St Louis, MO) for 8 hours; latter washed and cultured with regular complete media under various treatment conditions [22].

### Cyclin activity assay

The analysis of the Cyclin activity (Promega, Madison, WI), CDK2/CyclinA2 for G1/S activity and CDK1/CyclinA2 for G2/M activity was performed in the cell lysate under various assay conditions as per manufacturer’s instructions. The relative luminescence detection was performed using FilterMax F5 spectrophotometer (Molecular Devices, Sunnyvale, CA) and data obtained using instrument software. The data analysis was performed based on a standard curve obtained using the positive controls provided by the manufacturer (Promega).

### Enzyme linked immunosorbant assay (ELISA)

The secretory extracellular CXCL12 in the cell supernatant was quantitated by ELISA as per the manufacturer’s protocol (R&D Systems, Minneapolis, MN) [23, 24]. Similarly, AKT activity (AbCam, Cambridge, MA) and S6K1 activity (AbCam, Cambridge, MA) was analyzed by ELISA. Given the limitation of the detection, Quantification was performed with a standard curve using the manufacturer provided standards. Detection at 450 nm was performed using EMax Plus spectrophotometer and data analysis was carried out using software provided by the manufacturer (Molecular Devices, Sunnyvale, CA).

### CXCR4 membrane expression assay

CXCR4 expression was analyzed by flow cytometry as previously described [23]. Breifly, the CXCR4 protein was labeled by mouse anti-CXCR4 primary antibody (Santa Cruz Biotech, TX) in 1:20 dilution to a 200 μL final volume of cells (1 × 10^5^ cells/mL). Antibodies used for flow cytometry included anti-mouse-FITC (BD Biosciences, San Jose, CA), and the samples were latter analyzed using a FACS Calibur/LSRII flow cytometer (Becton-Dickinson, Franklin Lakes, NJ). Data were analyzed using BD FACSDiva software. Gates were set according to isotype controls.

### Nitric oxide/RNS assay

The analysis of the nitric oxide (NO), reactive oxygen and reactive nitrogen species (AbCam, Cambridge, MA) was performed in the cell lysate under various assay conditions as per manufacturer’s instructions [6]. The data analysis was performed based on a standard curve obtained using the positive controls provided by the manufacturer as previously described.

### Urea assay

The analysis of the urea in the (AbCam, Cambridge, MA) was performed in the cell supernatant under various assay conditions as per manufacturer’s instructions. The data analysis was performed based on a standard curve obtained using the positive controls provided by the manufacturer as previously described.

### Citrulline assay

The ELISA-based analysis of the citrulline in the (MyBiosource, San Diego, CA) was performed in the cell lysate under various assay conditions as per manufacturer’s instructions. Detection at 450 nm was performed using EMax Plus spectrophotometer and data analysis was carried out using software provided by the manufacturer (Molecular Devices, Sunnyvale, CA). The data analysis was performed based on a standard curve obtained using the positive controls provided by the manufacturer as previously described.

### Arginase-1 assay

The arginase activity analysis (AbCam, Cambridge, MA) was performed on the cell lysates under various assay conditions as per manufacturer’s instructions. Colorimetry detection at 570 nm was performed using EMax Plus spectrophotometer and data analysis was carried out using software provided by the manufacturer (Molecular Devices, Sunnyvale, CA). The data analysis was performed based on a standard curve obtained using the positive controls provided by the manufacturer as previously described.

### Gelatin zymography and MMP-2/9 activity

The MMP2 and MMP9 activity in the cell cultures was determined by gelatin zymography as previously described [18, 23, 25]. The protein concentration of the supernatants was determined using the BCA protein assay (Pierce, Rockford, IL), and 5 μg of supernatant proteins were resolved by non-reducing 10% SDS-PAGE through Novex Tris-Glycine gels containing 0.1% gelatin (Invitrogen, Carlsbad, CA). The gels were then developed as per manufacturer’s instructions (Invitrogen, Carlsbad, CA). The gelatinolytic activity of the MMPs, were quantitatively analyzed by the optical density of the bands using the Bio-Rad Universal Hood II (Hercules, CA). For MMP-9 enzymatic inhibition a specific inhibitor, 2-(*N*-Benzyl-4-methoxyphenylsulfonamido)-5-((diethylamino)methyl)-*N*-hydroxy-3-methylbenzamide (0.5 mM, ab142180, AbCam) was utilized [26]. The MMP2 and MMP9 enzymatic activity is further quantitated using gelatinase substrate activity (MMP-2/RPN2631, MMP-9/RPN2634, biotrack activity assay kit, GE Healthcare, Pittsburgh, PA) as per manufacturer protocols [23, 25]. Briefly, MMPs were captured by specific antibodies precoated microplate, the activity of which was eventually measured using chromogenic peptide substrate read at 405 nm. The concentration of active MMP is interpolated from a standard curve obtained using the manufacturer provided standard.

### Statistical analysis

Data are expressed as mean ± SEM from five independent studies. Statistical differences between means were analyzed using a paired or unpaired Student’s t test. A value of P less than 0.05 was considered significant. All data analysis was obtained using Origin 6 software (Origin Labs, Northampton, MA) or SPSS software, version 21 (IBM corporation, Armonk, NY).

## Results

### Identification of SIK3 as the key target mediating high salt synergized IL-17 induced breast cancer cell proliferation

**Fig 1:**
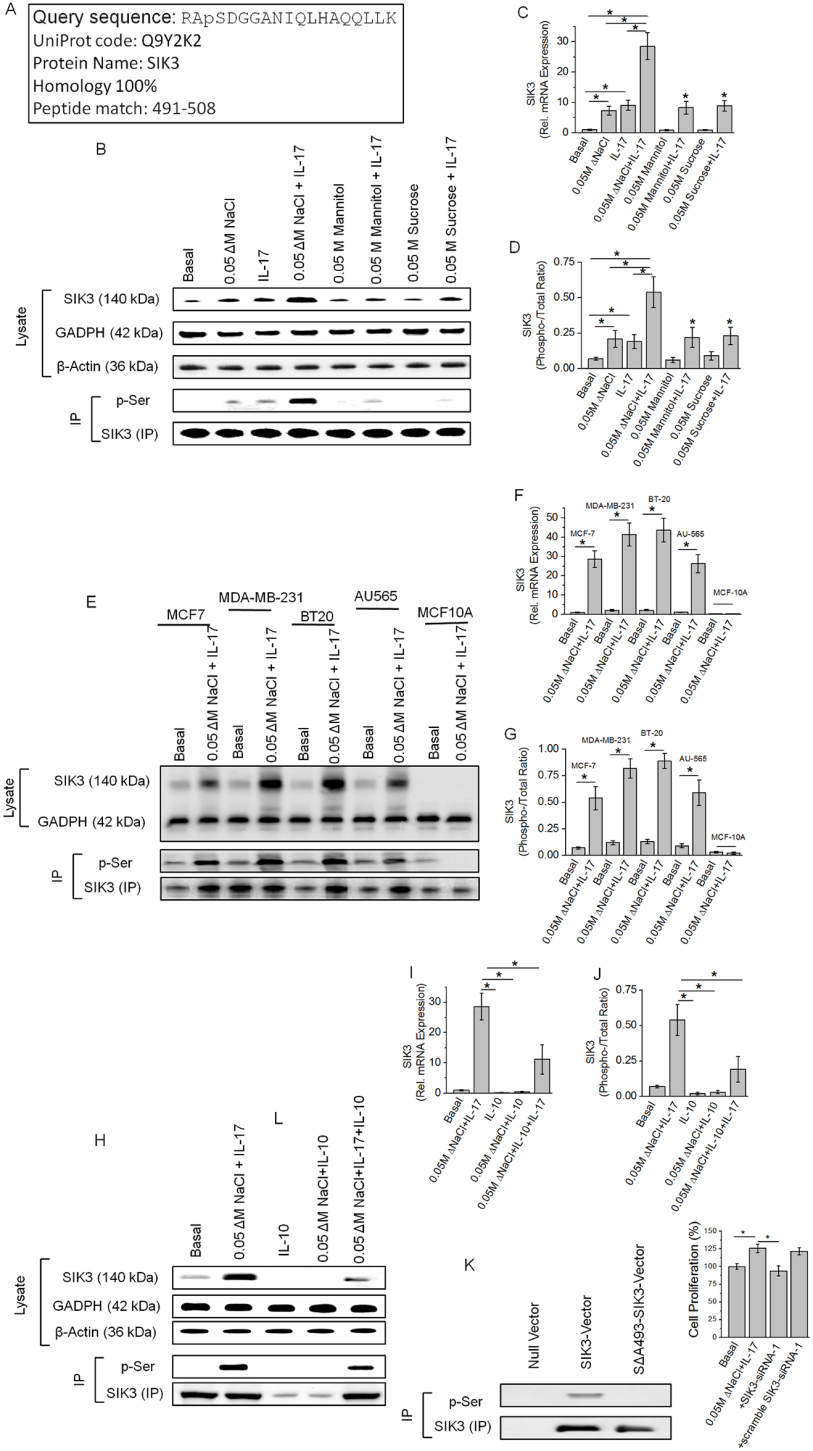
Identification of upregulation of SIK3 specifically following a high salt synergized IL-17 stimulation of breast cancer cells. (A) Identification of the 1.9 fold higher enriched phospho-peptide sequence following high salt induced stimulation of MCF-7 cells. The identified sequence demonstrated 100% homology to the SIK3 protein at the serine-493 residue. (B) Western blot analysis of SIK3 expression in total cell lysate. Immunoprecipitation of SIK3 is probed with phospho-serine antibody. As can be noted, SIK3 expression was upregulated following treatment with high salt (0.05 M NaCl) and IL-17 (0.1 ng/mL) individually, further, SIK3 expression was synergistically elevated following co-treatment with high salt and IL-17. Equimolar mannitol (0.05 M) and sucrose (0.05 M) were used as negative controls. (C) mRNA transcript expression of SIK3 by ΔΔcT method of quantitative real time polymerase chain reaction normalized for GADPH demonstrated a significant upregulation following high salt (7.3±1.4 fold) and IL-17 (9.1±1.7 fold), and co-treatment with both high salt and IL-17 induced a (28.6±4.4 fold) synergistically enhanced expression. (D) Densitometry quantitation of phospho-SIK3 in (B) demonstrated a synergistically enhanced phosphorylation of SIK3 by high salt and IL-17. (E-G) Expression of SIK3 following co-treatment with high salt and IL-17 in five breast tissue related cell lines (E) were used in our studies, of these, four breast cancer cells (MCF7, MDA-MB-231, BT20, AU565) and one non-malignant breast epithelial cell line (MCF10A); mRNA transcript analysis in 5 cell lines (F); densitometry quantitation of phosphorylated SIK3 in five cell lines (G). (H-J) Anti-inflammatory cytokine interleukin-10 (IL-10) inhibited the expression of SIK3 protein (H), mRNA transcript (I) and phosphorylation of SIK3(J). (K) To demonstrate the phosphorylation is specifically on Serine-493 of SIk3 we clone the SIK3 into MCF10A cell line and latter stimulated by co-treatment with high salt and IL-17. Of note, wild type MCF10A did not demonstrate SIK3 expression following co-treatment with high salt and IL-17 (E-G). Mutation of serine to alanine (SΔA493-SIK3) did not demonstration any binding with phospho-serine in SIK3 immunoprecipitate. (L) Cell proliferation of MCF-7 cells was inhibited following SIK3 siRNA treatment following co-treatment with high salt and IL-17. Of note, we have previously demonstrated that (Ref [6, 13]) co-treatment with high salt and IL-17 induced a 25% higher proliferation of MCF-7 cancer cells. The other three cancer cells MDA-MB-231 (29% higher), BT-20 (31% higher) and AU565 (24% higher) cell proliferation following co-treatment with high salt and IL-17, while non-malignant breast epithelial MCF10A cells did not demonstrate any enhanced proliferation following similar treatment conditions. All data represented as mean values ± SEM from four independent experiments. Student-t-test performed for statistical analysis (significance p<0.05).

We have previously performed a dose-response study with NaCl (Δ0 to Δ200 mM) [6, 13] and IL-17 (0–1000 ng/mL) [6, 14] and demonstrated that high salt at 50 mM above basal concentration (Δ0.05 M) along with sub-effective IL-17 (0.1 nM) is known to induce breast cancer cell proliferation and promote inflammatory responses. To specifically determine the molecular mechanisms mediating this response we have performed a binary phospho-proteomic analysis to determine key molecular targets upregulated following high salt synergized IL-17 inflammatory effect on MCF-7 breast cancer cells (Fig 1A). Our phospho-proteomic analysis (S1 Table) identified a unique salt sensitive kinase SIK3, salt inducible kinase-3 (Q9Y2K2; NCBI protein Id: NP_079440.2), a 1263 amino acid serine/threonine kinase, which has shown a specific phosphorylation at site ser-493 following co-treatment with NaCl (Δ0.05). We confirmed this enhanced expression of SIK3 with Westernblot analysis and quantitative reverse transcription polymerase chain reaction (qRT-PCR). As shown in Fig 1B and 1C, we have observed a 28 fold higher mRNA transcription and equivalent induction of expression of SIK3 protein following co-treatment with high salt and IL-17, the expression of which is higher than individual treatment of high salt or IL-17 separately. Equimolar (0.05 M) mannitol and sucrose were used as negative controls for the osmotic changes following treatment high salt. It is important to note that an equivalent ionic control cannot be used, as the other salts (lithium chloride, potassium chloride, rubidium chloride and cesium chloride) in the same group were found to be cytotoxic to the cells at just 5–10 mM concentrations in the culture media [27] (reference data obtained from chemical material safety data sheets). Immunoprecipitation of SIK3 with specific antibody and re-probing the immunopreciptate with phosho-serine antibody demonstrated specific phospho-serine phosphorylation of SIK3, suggesting that SIK3 is specifically phosphorylated following co-treatment with high salt and IL-17. To further determine if the SIK3 expression can be induced in other breast cancer cell lines, we have tested five breast-derived cell lines. Based on the standard receptor notation for breast cancer (estrogen receptor, ER; progesterone receptor, PR; Her2 receptor), we have used the following cell lines: MCF-7 (ER/PR double positive), MDA-MB-231 (triple negative), BT-20 (Triple negative), AU-565 (Her2 positive), MCF10A (non-malignant breast epithelial cell lines). As shown in Fig 1E, the four cancer cell lines MCF7, MDA-MB231, BT-20, and AU565 demonstrated upregulation of SIK3 expression following co-treatment with high salt and IL-17. While the non-malignant breast cell line MCF10A did not demonstrate SIK3 expression, suggesting SIK3 expression is specific to cancer phenotype. It is of interest to note that (Fig 1F) triple negative breast cancer cell lines (MDA-MB-231 and BT-20) known for their high treatment resistance have shown statistically significant (p<0.05) higher expression of SIK3 compared to receptor positive cell lines (MCF-7 and AU565). To determine if SIK3 is an inflammation-specific target we have performed experiments with treating the MCF-7 cells with anti-inflammatory cytokine IL-10. As shown in Fig 1H and 1I, treatment with anti-inflammatory cytokine IL-10 reduced the expression of SIK3 following co-treatment with high salt, thus strongly suggesting that SIK-3 is a salt-specific inflammatory marker. To confirm the phosphorylation site identified in our phospho-proteomic studies, we have transfected MCF10A cells with pcDNA3.1-SIK3 and treated with high salt and IL-17. As shown in Fig 1J, following co-treatment with high salt and IL-17 to non-malignant breast cell line MCF-10A which otherwise did not express SIK-3 under similar conditions, has demonstrated high expression of SIK3 protein following pcDNA3.1-SIK3 transfection. Importantly, these transfected MCF-10A cells have demonstrated phosphorylation of serine in SIK3 as probed with phospho-serine specific antibody, while transfection with alanine to serine mutant vector (ΔA493-SIK3) did not demonstration any phosphorylation. These data suggest that ser-493 on SIK-3 is specifically phosphorylated following co-treatment with high salt and IL-17. Furthermore, knockdown of SIK3 with siRNA (specificity of siRNA to silence SIK3 is demonstrated in latter section) has reduced the cell proliferation by upto 25%, supporting our previous claim that co-treatment of high salt with IL-17 induces upregulation of breast cancer cell proliferation. Taken together, these data suggest that SIK3 is a critical downstream factor specifically upregulated in breast cancer cells following co-stimulation with high salt and IL-17.

### mTOR2 and SIK3 are sequentially activated by IL-17 and salt, respectively

**Fig 2:**
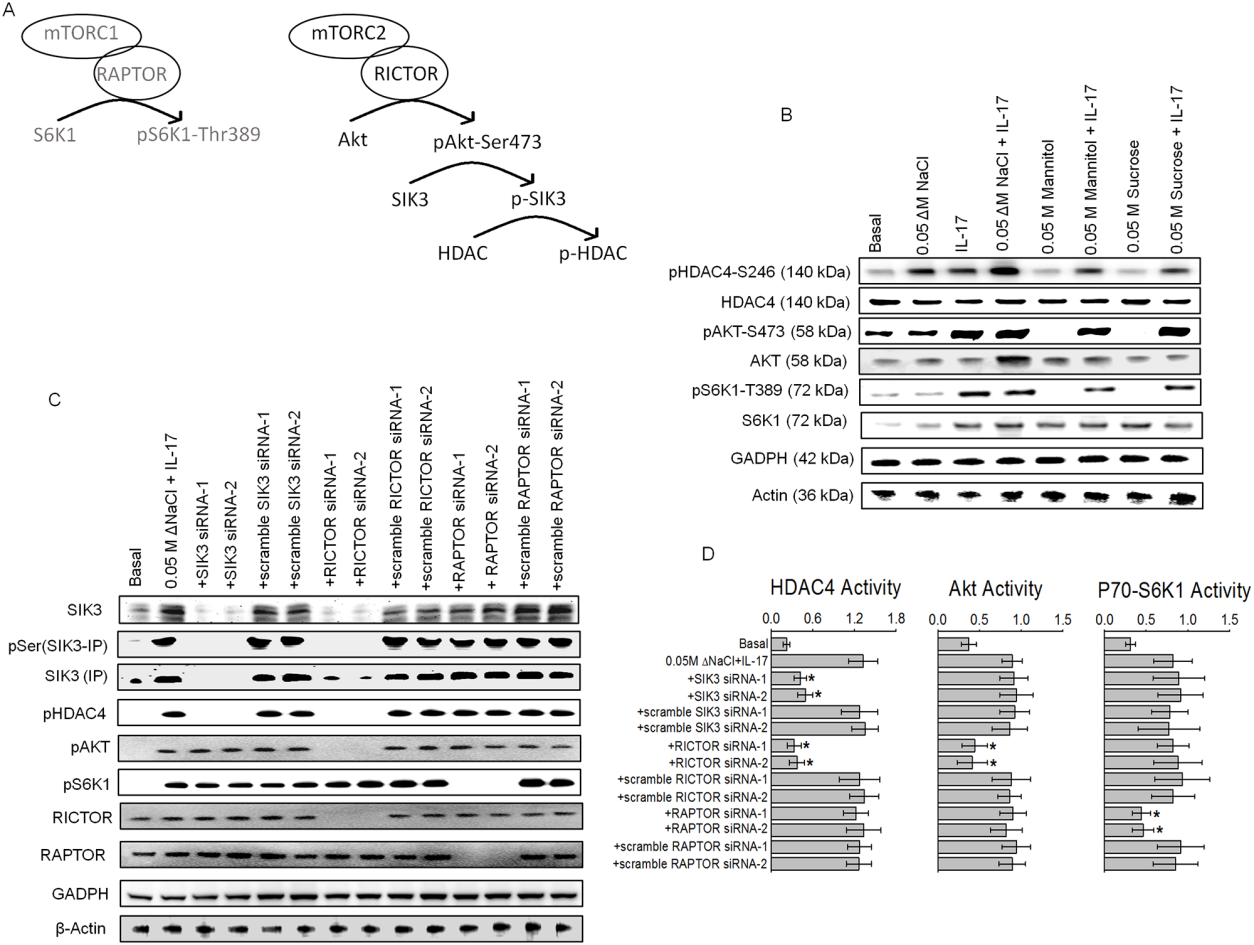
mTORC2 is an upstream upregulator of SIK3 expression and phosphorylation. (A) Schematic of the potential interplay between mTOR2 and SIK3. (B) Phosphorylation of HDAC4, AKT, and S6K1 the downstream targets for SIK3, mTOR1 and mTOR2, respectively, were analyzed following stimulation with high salt and IL-17, individually and combined by Western blot. (C) Phosphorylation of HDAC4, AKT, and S6K1 the downstream targets for SIK3, mTOR1 and mTOR2, respectively, were analyzed by Western blot following co-treatment with high salt and IL-17, and Knock down of SIK3, RAPTOR and RICTOR. Scramble siRNA of the original construct were used as controls. (D) Activity of HDAC4, Akt and S6K1 were analyzed by ELISA under various conditions mentioned in (C). Of note, HDAC4 activity was decreased following SIK3 and RICTOR knock down, while, mTOR2 downstream target AKT and mTOR1 downstream target S6K1 activity was decreased following RICTOR and RAPTOR, respectively. All data represented as mean values ± SEM from four independent experiments. Student-t-test performed for statistical analysis (significance p<0.05).

The mammalian target of rapamycin complexes (mTORC1 and mTORC2) mediate several central cellular regulatory functions including metabolism, growth and survival. Abnormal induction of mTOR signaling has been implicated in several cancers [28]. The mTOR compex is a highly conserved serine/threonine kinase, upregulated upon IL-17 mediated inflammatory responses [29]. Therefore, we studied the potential role of mTOR towards stimulation of SIK3 by inflammation mediated high salt and IL-17 injury. As mTOR complex has several constitutively expressed factors in several breast cancer cell lines, the modulation of these complexes upon inflammatory signal is best studied by inducible activation of its downstream pathway. Upon inflammatory activation (Fig 2A), mTORC1 phosphorylates its downstream target S6K1 at Threonine-389 [30], while mTORC2 phosphorylates its downstream target Akt at serine-473 [31]. As shown in Fig 2B, following co-treatment with high salt and IL-17 there is enhanced phosphorylation of HDAC4 a known downstream target for SIK3 [32]. The phosphorylation of HDAC4 demonstrated an additive effect with high salt and IL-17 over individual treatment with high salt or IL-17 alone. However, the phosphorylation of Akt and S6K1 responded to IL-17 alone and did not show much change with high salt treatment. To verify if SIK3 is a downstream target of mTOR complex, we have performed specific siRNA knockdown of RAPTOR, a key component of mTORC1 complex, or RICTOR, a key component of mTORC2 complex, and analyzed for the SIK3/HDAC4 phosphorylation. As shown in Fig 2C, knockdown of SIK3 by specific siRNA induced loss of SIK3 expression, phosphorylation of SIK3 and phosphorylation of HDAC4, with no effect on Akt and S6K1 phosphorylation. Importantly, knockdown of RICTOR, mTOR2 complex associated factor, by specific siRNA following co-treatment with high salt and IL-17 induced loss of SIK3 expression, phosphorylation of SIK3, phosphorylation of HDAC4 and phosphorylation of Akt, with no effect on S6K1 phosphorylation and thus suggesting that SIK3 is a downstream target of mTORC2. However, knockdown of RAPTOR, mTOR1 complex associated factor, by specific siRNA following co-treatment with high salt and IL-17 induced only loss of phosphorylation of S6K1 with no effect on SIK3 expression, phosphorylation of SIK3, phosphorylation of HDAC4 or phosphorylation of Akt, thus clarly indicating that mTORC1 has limited to no role in SIK3 signaling. These data assume significance in the light of our previous studies demonstrating high salt induced pro-Warburg like metabolic effect [33] and more importantly, mTORC2 has been strongly suggested to play a critical role in Warburg like glucose metabolism in cancer cells. We further confirm our finding by ELISA-based activity studies of HDAC4, Akt and S6K1 (Fig 2D) which further confirm that mTORC2 and SIK3 are sequentially activated by IL-17 and high salt, respectively, to potentially induce a synergistic inflammatory and cell proliferation effect in cancer cells.

### SIK3 mediated high salt induced cell cycle progression through release of G1/S arrest

**Fig 3:**
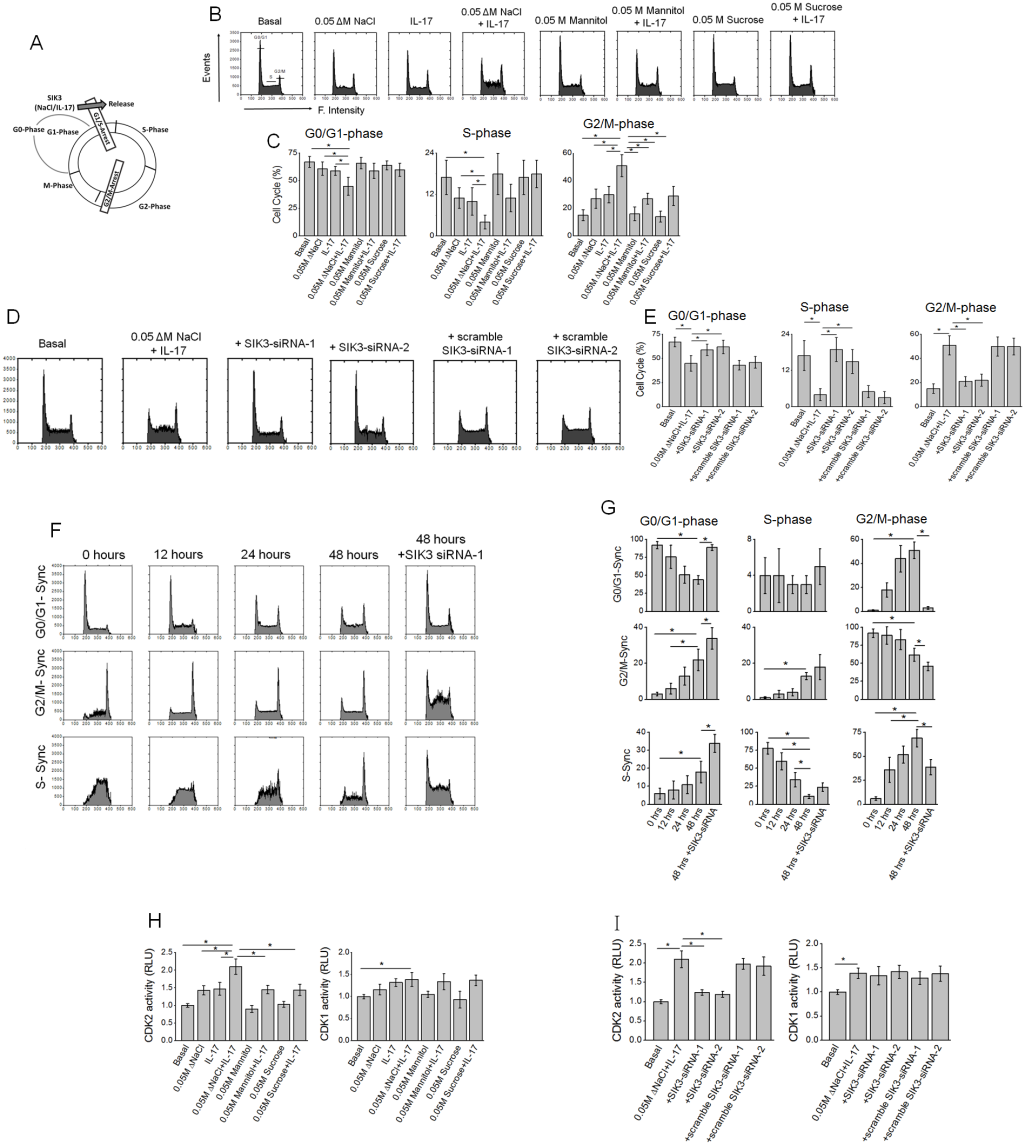
High salt synergized with IL-17 to induce SIK3 mediated G0/G1-release in cell cycle. (A) Schematic of cell cycle and potential impact of co-treatment with high salt and IL-17. (B) Flow cytometry analysis of cell cycle following treatment with high salt and IL-17, individually and combined. (C) Relative percentage of cells (%) in G0/G1-, S-, and G2/M-phase following various treatment conditions mentioned above. (D-E) Impact of SIK3 knockdown by siRNA on the three cell cycle phases. (F-G) Temporal Synchronization cell cycle studies following initial chemical synchronization to either G0/G1-, S-, or G2/M-phase and followed by co-treatment with high salt and IL-17 with cell cycle analysis done at 0, 12, 24 and 48 hour time points. The last panel is with cells initially synchronized to various cell cycle phases followed by co-treatment with high salt and IL-17 for 12 hours, followed by siRNA transfection (SIK3 or scramble) at 12 hours and followed by co-treatment with high salt and IL-17 for another 36 hours and data analyzed at 48 hour-time point. (H) Analysis of G0/G1-phase release mediating CDK2 and G2/M-phase release mediating CDK1 activity at 48 hour time point following treatment with high salt and IL-17 individually and combined. As can be noted, co-treatment with high salt and IL-17 induced CDK2 activity and release from G0/G1-phase arrest of cell cycle. (I) Analysis of G0/G1-phase release mediating CDK2 and G2/M-phase release mediating CDK1 activity following siRNA knockdown of SIK3 (as mentioned under F-G)and co-treatment with high salt and IL-17. All data represented as mean values ± SEM from four independent experiments. Student-t-test performed for statistical analysis (significance p<0.05).

To determine the role of SIK3 on high salt and IL-17 synergized proliferative effect on cancer cells, we studied the effect of SIK3 on cell cycle phases following various treatment conditions. As shown in Fig 3A and 3B, treatment of cancer cells with high salt and IL-17 induced an increased percentage of cells in G2/M phase (from 16% under basal conditions to 51% following high salt plus IL-17 treatment). This suggests that high salt and IL-17 co-treatment induced a cell proliferation through release of G1/S check-point arrest. Further, knockdown of SIK3 by specific siRNA (Fig 3C and 3D) reversed this effect on progression to G2/M phase. To specifically study for the individual check points, we have performed cell cycle synchronization studies, by cells chemically synchronized to various phases namely, G0/G1-, S- and G2/M-phases. As shown in Fig 3E, synchronization of cells to G0/G1-phase and latter stimulation with high salt and IL-17 induced a 48% progression of cells to G2/M phase (compared to 51% under unsynchronized conditions) with 48 hours following synchronization. This suggests that high salt plus IL-17 was able to effectively release the G1/S checkpoint arrest. However, similar stimulation conditions in cells synchronized to G2/M phase (Fig 3G) did not demonstrate a major shift in cell cycle to G0/G1-phase (22% in G0/G1 synchronization versus 45% under unsynchronized conditions). These G2/M synchronization studies suggest that cells were trapped in G2/M-phase and high salt plus IL-17 could not effectively release the G2/M-checkpoint arrest. We confirmed these finding by studying the CDK2 and CDK1 activity assay. The cyclin dependent kinase-2 (CDK2) activity is known to induce G1/S transition, while cyclin dependent kinase-1 (CDK1) activity is known to induce G2/M transition. As shown in Fig 3I, high salt and IL-17 induced CDK2 activity which was significantly diminished following SIK3 knockdown with siRNA under similar treatment conditions. While we observed only minimal changes in CDK1 activity suggesting no significant impact on G2/M-checkpoint arrest was observed. Taken together, these data strongly suggest that high salt and IL-17 exert its cell proliferative effect through SIK3 mediated release of G1/S-phase arrest.

### SIK3 induced pro-inflammatory arginine metabolism and release of reactive nitrogen species

**Fig 4:**
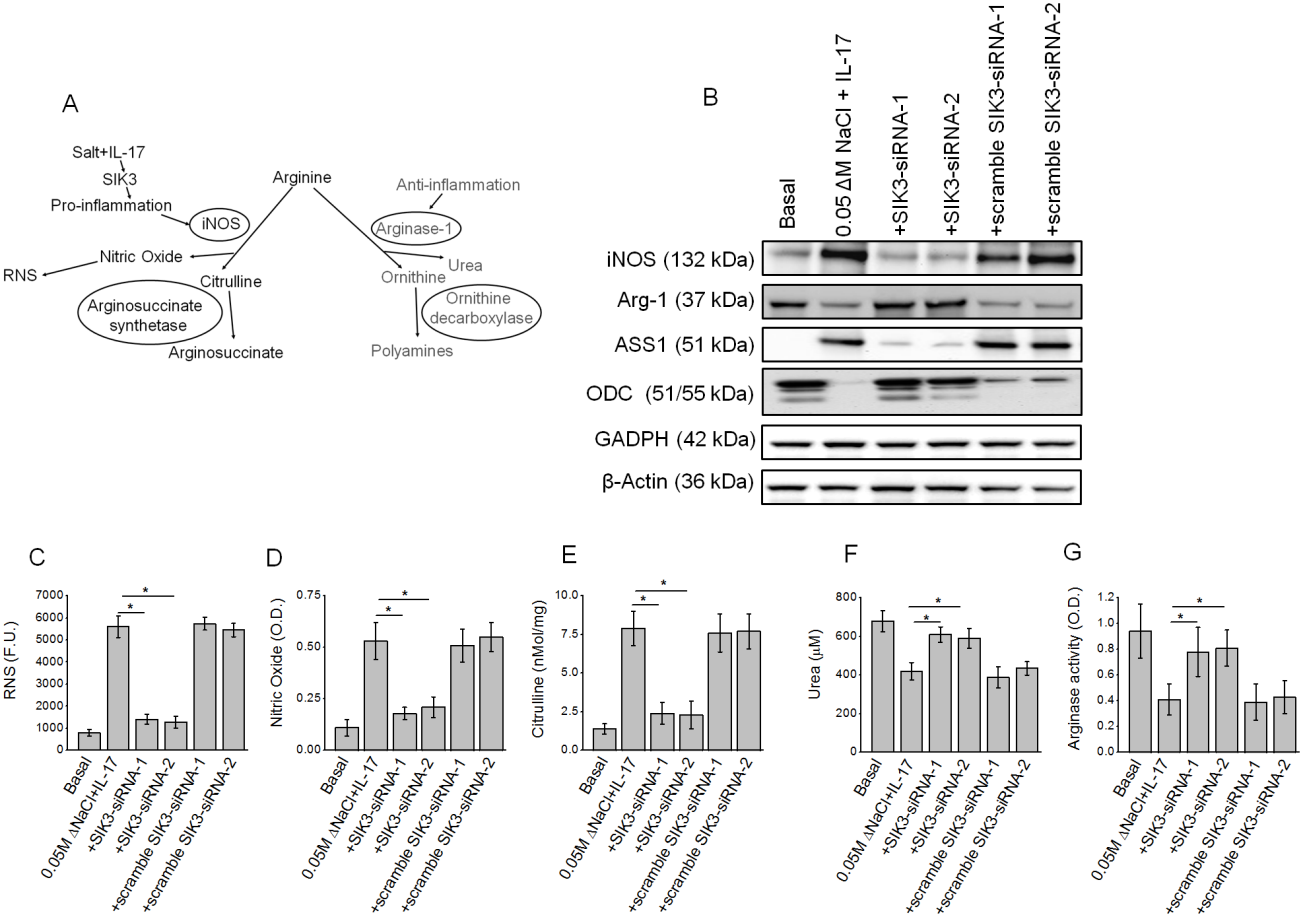
High salt synergized with IL-17 to induce SIK3 mediated pro-inflammatory nitric oxide release components of arginine metabolism while suppressing anti-inflammatory components of arginine metabolism. (A) Schematic of arginine metabolism under potential pro-inflammatory and anti-inflammatory stimulus conditions. (B) Western blot analysis of the pro-inflammation mediating enzymes inducible nitric oxide synthetase (iNOS) and arginosuccinate synthetase (ASS-1); and anti-inflammation mediating enzymes arginase-1 (Arg-1) and ornithine decarboxylase (ODC) following co-treatment with high salt and IL-17 along with SIK3 knockdown. (C-G) production of reactive nitrogen species (C), nitric oxide (D), citrulline (E), urea (F) and arginase activity (G) following co-treatment with high salt and IL-17, along with SIK3 knock down. All data represented as mean values ± SEM from four independent experiments. Student-t-test performed for statistical analysis (significance p<0.05).

We have previously demonstrated that following co-treatment with high salt and IL-17 synergized there was increased formation of nitric oxide and reactive nitrogen species suggesting that these treatment conditions induced an inflammatory response in breast cancer cells [6]. To determine if SIK3 is directly involved in these inflammatory responses following co-treatment with high salt and IL-17, we studied the formation of inflammatory metabolites of arginine metabolism were analyzed. As shown in Fig 4A–4C, co-treatment with high salt and IL-17 induced upregulation of reactive nitrogen species formation (7.1 fold), nitric oxide release (4.8 fold), citrulline (5.9 fold) above basal culture conditions. Importantly, this increase was abrogated with SIK3 knockdown following co-treatment with high salt and IL-17. These data suggest that high salt induced inflammatory reactive nitrogen species response is mediated through SIK3. Conversely, the anti-inflammatory arginine metabolic pathway is downregulated (Fig 4D and 4E) as evidenced by decrease in urea formation (1.6 fold decrease)and arginase-1 activity (2.3 fold decrease) following co-treatment with high salt and IL-17 as compared to basal conditions. This is further supported by Western blot analysis of the key enzymes in arginine pathway. As shown in Fig 4F, following co-treatment with high salt and IL-17 there is enhanced expression of pro-inflammatory inducible nitric oxide synthetase (iNOS) and Arginosuccinate synthetase (ASS-1), which was efficiently decrease following SIK3 knockdown under similar treatment conditions. Conversely, under these conditions, there was downregulation of anti-inflammatory arginase-1 and ornithine decarboxylase. Taken together, these data strongly suggest that high salt synergizes with IL-17 to induce formation of inflammatory reactive nitrogen species and SIK3 play a critical role in mediating this inflammatory process.

### SIK3 induced expression of tumor metastatic CXCR4 through MMP-9 activation

**Fig 5:**
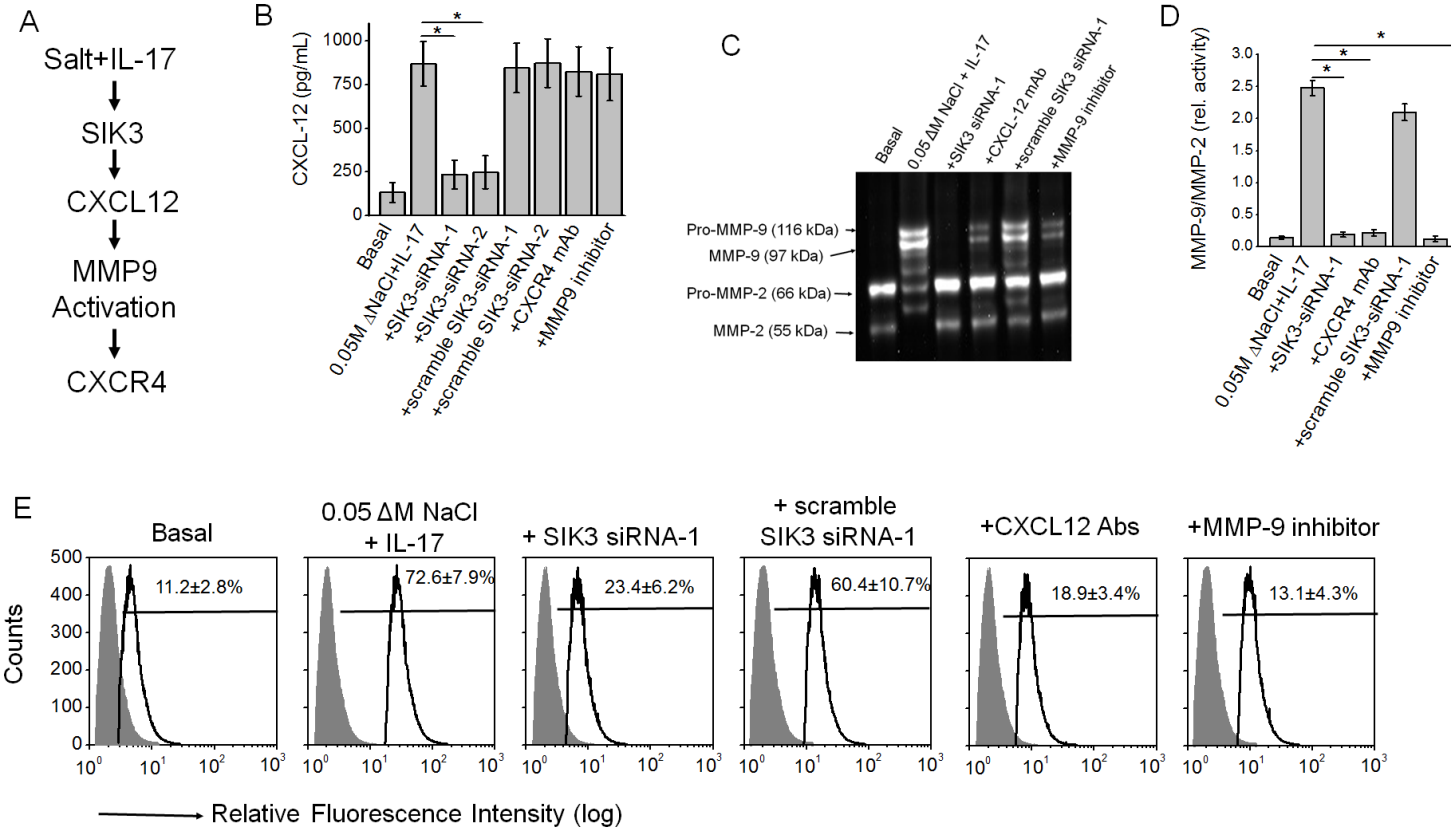
High salt synergized with IL-17 to induced SIK3 mediated expression of tumor metastatic CXCR4 through MMP-9 activation. (A) Schematic of the metastasis mediating CXCL12/CXR4. (B) ELISA-based analysis in the cell supernatant for the CXCL12 expression following co-treatment with high salt and IL-17 along with SIK3 knockdown or MMP-9 inhibitor (10 μM, 2-(*N*-Benzyl-4-methoxyphenylsulfonamido)-5-((diethylamino)methyl)-*N*-hydroxy-3-methylbenzamide, ab142180, AbCam, Cambridge, MA) or CXCR4 blocked with specific monoclonal antibody (Santa Cruz, Dallas, TX). (C) Gelatin Zymography to analysis the MMP-9/-2 activity under the above mentioned treatment conditions. (D) Ratio of inducible MMP-9 to constitutive MMP-2 activity under above mentioned treatment conditions. (E) Flow cytometry-based analysis in the membrane localization of the CXCR-4 following co-treatment with high salt and IL-17 along with SIK3 knockdown or MMP-9 inhibitor or CXCL12 blocked with specific monoclonal antibody (Santa Cruz, Dallas, TX). All data represented as mean values ± SEM from four independent experiments. Student-t-test performed for statistical analysis (significance p<0.05).

The chemokine CXCL12 and its receptor CXCR4 has been demonstrated to cause tumor progression and metastasis. We have previously shown that inflammatory cytokines induce CXCR4 expression through CXCL12 mediated MMP9 activation pathway [23]. As metastasis is a one of the hallmarks of cancer proliferation, we studied the potential effect of SIK3 on the expression of CXCR4 chemokine receptor, an adhesion and tumor metastatic factor. Further, CXCR4 is stimulated by secretory inflammatory chemokine CXCL12. We studied the expression of CXCL12 in the supernatant media following stimulation with high salt and IL-17. As shown in Fig 5B, following treatment with high salt and IL-17 there is increased secretion of CXCL12 (869 ± 128 pg/mL) over basal conditions (132 ± 56 pg/mL). This increase was inhibited by SIK3 siRNA, suggesting that SIK3 plays a critical role in CXCL12 expression. However, neither CXCR4 monoclonal antibodies (mAb) nor MMP-9 inhibitor induce any inhibition of CXCL12 expression. Previous studies have shown CXCL12 induces MMP9 activation. Consistent with these studies. MMP9 gelatin zymography studies (Fig 5C and 5D) have demonstrated that high salt and IL-17 induced MMP-9 activation which was inhibited by SIK3 siRNA (13 fold), CXCL12 mAb (11 fold), and direct small molecule MMP9 inhibitor (20 fold). Several studies in literature have demonstrated that MMP9 activation is needed for membrane expression of CXCR4, we have analyzed for the CXCR4 membrane expression following treatment with high salt and IL-17. As shown in Fig 5E, flow cytometry analysis has demonstrated that membrane expression of CXCR4 was upregulated from 8.7±1.4% under basal conditions to 74.4±9.3% following co-treatment with high salt and IL-17. Importantly, this CXCR4 expression was diminished following SIK3 knock down (10.9±2.2%), CXCL12 mAb treatment (16.2±3.7%) and MMP-9 inhibition (18.6±4.1%) following similar high salt and IL-17 treatment conditions. Taken together, these data clearly demonstrate that SIK3 mediates pro-metastatic CXCR4 expression following high salt and IL-17 induction to cancer cells.

## Discussion

Recent understanding has established a strong correlation between chronic inflammation and cancer [34]. A multi-factorial etiology including, chemical carcinogens, infections and diet have been suggested to play an important role in chronic inflammation. Several epidemiological studies have demonstrated that high salt (sodium chloride) diet correlated with several chronic inflammatory diseases [35, 36]. Other than in gastric cancers, to date, there is no evidence of direct correlation between high salt diet and human cancers [37]. However, importantly, sodium-MRI studies have demonstrated a sodium concentration in the breast cancer microenvironment [8]. Previous studies in our laboratory have demonstrated that high salt induced cancer cell proliferation and inflammatory reactive oxygen and nitrogen species [6]. To determine the molecular events induced by high salt in the inflammatory tumor microenvironment, we have performed phospho-proteomic studies on MCF-7 cancer cells following stimulation with high salt and sub-effective inflammatory cytokine (IL-17) stimulation. We have identified a unique salt-specific molecule, salt-inducible kinase-3 (SIK3), whose phosphorylation at Ser-493 is upregulated following stimulation under above mentioned conditions.

Salt-inducible kinases (SIK) are originally studied as central molecule controlling adrenocorticotrophic hormone mediated gene expression to maintain sodium/potassium homeostasis [38]. Of the three isoforms of SIK, the SIK1 is shown to be expressed in adrenal cortex, SIK2 is determined to be more specific to adipose tissue, while SIK3 is shown to be ubiquitously expressed in all tissues. Importantly SIK3 is overexpressed in human ovarian cancers [39]. In line with this literature evidence, we demonstrate specific upregulation of SIK3 following stimulation with high salt on cancer cell lines. Interestingly, we have shown higher expression of SIK3 in triple negative breast cancer cell lines (MDA-MB-231 and BT-20) which are known to exert treatment resistance. Triple negative breast cancer is more common in women of African-American decent, while receptor positive cancers are more common in women of European-American decent [40]. Identification of SIK3 could possibly offer novel therapeutic targets to treat triple negative breast cancers. Importantly, MCF10A cell lines (non-malignant breast epithelial cell line) did not show significant SIK3 expression under high salt treatment conditions, possibly suggesting SIK3 expression requires other cancer-associated gene machinery.

SIK3 is shown to be homologous to AMP-activated kinases along with a coordinated function with mammalian target of rapamycin (mTOR) complex [41, 42]. The mTOR complex has been demonstrated to exert a pro-cancerous effect [28]. This complex is considered to be activated under stress and by inflammatory cytokines. In line with these observations, following sub-effective IL-17 treatment we saw an activation of both mTORC1 and 2, but specifically mTORC2 exert a downstream cascading effect to phosphorylate SIK3 and its substrate HDAC4. Masui et al have recently demonstrated that mTORC2 enhances Warburg-like glucose metabolism and drug resistance in cancer cells [33]. Previous studies from our lab have demonstrated that high salt enhances Warburg-like metabolism in cancer cells [13]. Our current studies along with previous observations potential suggest a mTORC2/ SIK3 cascade plays an important role in Warburg metabolism and enhances cancer proliferations. Further metabolomics studies in this direction are needed to demonstrate the role of SIK3 in Warburg metabolism. Studies by Walkinshaw et al have demonstrated that tumor suppressor kinase LKB1 is an upstream modulator of SIK3 phosphorylation in HEK293 and HeLa cell lines [32]. Possibly both mTORC2 and LKB1 could have a synergistic or mutual exclusive effect depending on the type of cancer, which needs to be studied in multiple cancer cells.

Studies by Charoenfuprasert et al in ovarian cancer cells have demonstrated that SIK3 promotes G1/S-cell cycle progression [39]. In these studies the authors have demonstrated that SIK3 upregulates expression of cyclinD and E. In agreement with these findings, our current studies demonstrate an enhanced G1/S phase cell cycle in breast cancer cells. High salt synergized IL-17 has demonstrated upregulation of G1/S promoting CDK2 activity with no effect on G2/M promoting CDK1 activity. Further synchronization of cell cycle in G2 phase along SIK3 knock down significantly abrogated the cell cycle progression following high salt and IL-17 stimulation, suggesting that SIK3 exerts its cancer proliferation effect through release of G1/S arrest. Furthermore, our data are in agreement with studies by Chen et al., who using time-lapse microscopy have demonstrated that SIK3 depletion resulted in aberrant mitotic spindle formation and decreased mitotic index in HeLa cells [43]. Our current data along with evidence from other laboratories clearly suggest that SIK3 could offer a novel molecular target for anti-mitotic drug development strategies. Further, SIK3 inhibitors could be coupled with drugs that target other known cell division promoters like Aurora kinases (AurK-A; AurK-B) and cAMP kinases.

Chronic inflammation is strongly correlated with cancer. Previously we have demonstrated that high salt synergizes with IL-17 to induce reactive nitrogen and oxygen species [6]. In our current studies we demonstrate that SIK3 plays a direct role in mediating this inflammatory insult. Arginine pathway is considered a major modal point for production of nitric oxide and reactive nitrogen species [44]. We have shown that the pro-inflammatory enzymes iNOS and ASS-1 are upregulation through SIK3 mediated inflammatory events resulting from high salt stimulation while anti-inflammatory Arg-1 and ODC are downregulated. Metastasis and spreading to other organs from original site is one of the hallmarks of cancer cells [45]. Several lines of evidence have conclusively suggested that expression of chemokine CXCL12 and its specific receptor CXCR4 on cancer cells promote metastasis [46]. To date, there is very limited evidence on the role of SIK3 in cancer metastasis. We show that the metastasis specific inflammatory molecules are specifically upregulated by SIK3 in breast cancer cell lines. It is interesting to note that studies by Lombardi et al, have also demonstrated that SIK3 inhibition results in induction of anti-inflammatory phenotype of human myeloid cells, which are a part of human innate immune responses [47]. However, conversely, studies by Kim et al, have demonstrated that in macrophages SIK3 specifically plays a role in inhibiting inflammatory TLR-4 mediated signaling [48]. Furthermore, it is important to note that in the tumor microenvironment, while there is increased pro-inflammatory RNS/ROS production by tumor cells, there is an opposite anti-inflammatory macrophage phenotype surrounding the tumor cells to suppress immune elimination of cancer cells [49]. These data suggest that SIK3 might exert different roles based on the cell type and cell-differentiation status of individual cell phenotypes cumulatively acting to promote pro-tumor effect.

While, our current study is limited due to heavy reliance on cell based studies, nevertheless, our studies based of five cell lines provide mechanistic basis to perform future murine and other small animal cancer based experiments to study the precise effect of salt in tumor microenvironment. Our studies suggested that majority of the expressed SIK3 is predominantly sequestered in the cytoplasmic fragment of the cell (Fig 1), suggesting multiple cytoplasmic binding partners and downstream nuclear transcription factors down stream of SIK3. To date, HDAC4 is the only well-characterized DNA modulating factor under downstream control of SIK3. Future studies based on transcription factor array and binding partner analysis by ChIP-seq will help determine specific signaling events mediated by SIK3. While our current experiments were limited to breast cancer studies, we think the mechanisms of SIK3 signaling might be related to all solid tumors. We have currently shown that SIK3 is specifically expressed in breast cancer cells over normal breast cells, and also studies by others in ovarian cancer have confirmed SIK3 as a potential biomarker, drug target and/or tumor associated antigen, which offers further venues of research in this direction. In conclusion, we report upregulation of SIK3 a novel serine/threonine kinases specifically in the in vitro high salt tumor microenvironment with future application cancer diagnosis and drug therapy.

## Supporting information

S1 Table Binary phospho-proteomic analysis to determine changes in the phosphorylation between heavy-labeled MCF-7 cells treated under high salt (Δ0.05 M NaCl) and light-labeled MCF-7 cells cultured under basal conditions. A cut-off heavy to light ratio (H/L) of >1.8 is considered significant.
(XLSX)

## Abbreviations

ASS-1: Arginosuccinate synthetase-1
IL: interleukin
iNOS: inducible nitric oxide synthetase
mTORC: mammalian target of rapamycin complex
NaCl: Sodium chloride/salt
ODC: ornithine decarboxylase
SIK3: Salt inducible kinase-3
